# Insight Into the Metabolomic Characteristics of Post-Transplant Diabetes Mellitus by the Integrated LC-MS and GC-MS Approach- Preliminary Study

**DOI:** 10.3389/fendo.2021.807318

**Published:** 2022-01-18

**Authors:** Min Wang, Jie Xu, Na Yang, Tianqi Zhang, Huaijun Zhu, Jing Wang

**Affiliations:** ^1^ Department of Pharmacy, Nanjing Drum Tower Hospital, The Affiliated Hospital of Nanjing University Medical School, Nanjing, China; ^2^ Physical and Chemical Department, Nanjing Center for Disease Control and Prevention, Nanjing, China; ^3^ College of Pharmacy, Shaanxi University of Chinese Medicine, Xianyang, China

**Keywords:** post-transplant diabetes mellitus, metabolomic characteristics, LC-MS, GC-MS, potential mechanism

## Abstract

Post-transplantation diabetes mellitus (PTDM) is a common metabolic complication after solid organ transplantation, which not only results in elevated microvascular morbidity, but also seriously impacts graft function and recipient survival. However, its underlying mechanism is not yet fully understood. In this study, an integrated liquid chromatography- mass spectrometry (LC-MS) and gas chromatography-mass spectrometry (GC-MS) based-metabolomics approach was adopted to dissect the metabolic fluctuations and deduce potential mechanism associated with PTDM. 68 adult liver transplant recipients were recruited and classified as 32 PTDM and 36 non-PTDM subjects. PTDM group and non-PTDM group were well matched in gender, age, BMI, family history of diabetes, alcohol drinking history, ICU length of stay and hepatitis B infection. Peripheral blood samples from these recipients were collected and prepared for instrument analysis. Data acquired from LC-MS and GC-MS demonstrated significant metabolome alterations between PTDM and non-PTDM subjects. A total of 30 differential metabolites (15 from LC-MS, 15 from GC-MS) were screened out. PTDM patients, compared with non-PTDM subjects, were characterized with increased levels of L-leucine, L-phenylalanine, LysoPE (16:0), LysoPE (18:0), LysoPC (18:0), taurocholic acid, glycocholic acid, taurochenodeoxycholic acid, tauroursodeoxycholic acid, glycochenodeoxycholic acid, glycoursodeoxycholic acid, etc, and with decreased levels of LysoPC (16:1), LysoPC (18:2), LysoPE (22:6), LysoPC (20:4), etc. Taken collectively, this study demonstrated altered metabolites in patients with PTDM, which would provide support for enhancing mechanism exploration, prediction and treatment of PTDM.

## 1 Introduction

Solid organ transplantation (SOT), with more than 110,000 transplantations performed worldwide annually (1), is the treatment of choice for patients with end-stage organ failure. The short-term outcome of SOT improved remarkably due to advances in organ preservation (2), surgical techniques (3), immunosuppression regimens (4) and so on. However, metabolic complications, such as diabetes mellitus, hypertension and dyslipidemia, severely impact the long-term survival (5, 6).

Diabetes mellitus after SOT, defined as post-transplantation diabetes mellitus (PTDM), is considered to be a variant of type 2 diabetes mellitus (T2DM). PTDM is formally diagnosed at least 45 days post-transplantation and has a sudden onset within the first year post-transplantation (7). The reported prevalence of PTDM varies from 30% to 40% in liver recipients, 10% to 40% in renal recipients and 20% to 40% in other SOT recipients (8). PTDM is one of the major risk factors for diabetes-associated microvascular complications and infections, contributing to 1.63 times higher risk of graft failure and 1.87 times higher risk of mortality in SOT recipients (9).

Despite the prevalence and unfavorable outcomes associated with PTDM, the mechanism underlying PTDM is not entirely known. Over the past few decades, scientists devoted to evaluate factors affecting PTDM occurrence, such as age, gender, hepatitis infection, family history of type II diabetes mellitus, body mass index and immunosuppressive agents (10–12). Since PTDM is a serious frequent metabolic complication characterized by hepatic glucose overproduction, insulin hyposecretion and resistance, it is reasonably assumed that many metabolites and pathways are quite likely to be interrupted and play a critical role in the whole-body metabolic dysfunction. Thus, the comprehensive measurement and characterization of altered metabolites could give insights into the metabolic mechanism of PTDM.

Metabolomics is an invaluable tool for reflecting a series of biological processes underlying metabolic homeostasis and their complex association with peculiar disease, lifestyle, or genetic modifications, etc (13). Compared to targeted metabolomics focusing on well-defined metabolites, untargeted metabolomics aims at the qualitative or quantitative monitoring of all low-molecular-weight metabolites in a biological fluid and has been widely used to discover specific metabolic patterns of diseases (14). A range of analytical platforms including gas chromatography-mass spectrometry (GC-MS) (15), liquid chromatography-MS (LC-MS) (16), nuclear magnetic resonance (NMR) spectroscopy (17) and direct infusion MS (18) have been widely applied in metabolomics area. Among these, GC-MS and LC-MS are the two most powerful and commonly used analytical techniques owing to their high resolution of the chromatographic system, high sensitivity of MS detector and wide detection magnitude during the qualification and quantification of metabolites. Moreover, since no single analytical platform can cover the entire metabolome in a biological sample, the integration of GC-MS and LC-MS would serve as an appropriate strategy to capture a broader spectrum of metabolites (19, 20).

In this study, we aimed to primarily screen out the differentially expressed metabolites in PTDM and explore its potential pathophysiological mechanism by analyzing the metabolomic characteristics of PTDM recipients with the aid of the integrated liquid chromatography- mass spectrometry (LC-MS) and gas chromatography-mass spectrometry (GC-MS) based-metabolomics. For the first time to our knowledge, the metabolic profiles involved in PTDM were explored, which would provide novel insights into the underlying mechanisms of PTDM from the perspective of metabolomics.

## 2 Materials and Methods

### 2.1 Patients and Sample Collection

Adult (age ≥ 18 years) liver transplant recipients who had undergone primary liver transplantation between July 2019 and June 2020 at the Affiliated Drum Tower Hospital of Nanjing University Medical School, China were enrolled in this study. Patients were excluded if they were followed up less than one year after transplantation, underwent ABO incompatible transplantation, received a multi-organ transplantation, had diabetes mellitus prior to transplantation or developed acute rejection. The receipts received a standard triple-drug immunosuppression regimen including tacrolimus, mycophenolate mofetil and corticosteroids.

The experimental protocol was reviewed and approved by the Ethics Committee of the Affiliated Drum Tower Hospital of Nanjing University Medical School (No. 2020-053-01). Signed informed consent was exempted due to the deidentified data provided to researchers and residual biosamples used.

According to the International Consensus Meeting on PTDM (7), PTDM is diagnosed at least 45 days post-transplantation using the American Diabetes Association (ADA) criteria for type 2 diabetes mellitus: with symptoms of diabetes plus random plasma glucose ≥ 200 mg/dL (11.1 mmol/L) or fasting plasma glucose ≥126 mg/dL (7.0 mmol/L) or 2-h plasma glucose after an oral glucose ≥200 mg/dL (11.1 mmol/L) during an OGTT or glycated hemoglobin (HbA1c) ≥6.5%. In this study, 32 and 36 recipients were assigned into the PTDM group and the non-PTDM group, respectively. Peripheral blood samples from these recipients were collected after overnight fasting at time of PTDM diagnosis and centrifugated at 1760 g for 10 min to prepare plasma. All the plasma samples were then divided into aliquots and stored at -80°C until analysis.

### 2.2 LC-MS Based-Metabolomics

#### 2.2.1 Sample Preparation for LC-MS Based-Metabolomics

Plasma was thawed in a refrigerator at 4°C and thoroughly vortexed with seven times pure ice-cold acetonitrile for 5 min. The mixture was then centrifuged two times at 18407 g for 10 min at 4°C prior to injection into LC-MS system.

#### 2.2.2 LC-MS Spectral Acquisition

Chromatographic separation was achieved on Shimadzu Prominence series ultra-fast liquid chromatography (UFLC) system equipped with Phenomenex Kinetex C18 column (100×2.1 mm, 2.6μm; Phenomenex, Torrance, CA, USA) and a guard column, SecurityGuard ULTRA cartridge UHPLC C18 for 2.1 mm ID column (Phenomenex, Torrance, CA, USA). The column and autosampler were set at 40°C and 4°C, respectively. The gradient elution involved a mobile phase consisting of acetonitrile (mobile phase A) and 0.1% formic acid (mobile phase B) with a gradient program as follows: 5%-95% A, 0-20 min and 95%A, 20-23 min. The mobile phase was directly delivered into mass spectrometer at 0.4 mL/min, and the injection volume was 5 μL.

Mass spectrometry was performed on an ion trap/time-of-flight hybrid mass spectrometry with an electrospray ionization (ESI) source (IT/TOF-MS, Shimadzu, Japan). The mass spectrometer was operated simultaneously in positive and negative electrospray ionization modes by switching the interface voltage between 4.5 kV and -3.5 kV. The other parameters were set as follows: curved desorption line (CDL) temperature, 200°C; heat block temperature, 200°C; microchannel plate detector voltage, 1.65 kV; nebulizer gas (N_2_), 1.5 L/min; drying gas (N_2_), 10.0 L/min; collision energy, 10%, 30% and 60%. MS/MS analyses were conducted in data dependent acquisition, in which precursor ions are serially fragmented to generate their corresponding product-ion spectra. Product-ion spectra were acquired automatically in advance for a large number of ions. Furthermore, if the MS/MS information of the selected discriminating variables was missing, the product ion spectrum for these variables were acquired independently in manual mode. External calibration using the sodium trifluoroacetate was adopted to regulate the MS and MS/MS data.

### 2.3 GC-MS Based-Metabolomics

#### 2.3.1 Sample Preparation for GC-MS Based-Metabolomics

The plasma was prepared with a two-step derivatization procedure, that is, alkylation and silylation, according to previous reports with a few modifications (21, 22). Briefly speaking, a 10 µL aliquot of plasma was thoroughly vortexed with ten times methanol followed by centrifuged at 18047g at 4°C for 10 min in two cycles. Then 80 μL supernatant was transferred to a brown glass vial and oximated with 25 μL methoxyamine hydrochloride (10 mg/mL in pyridine) at 4°C for 90 min. Finally, the mixture was vacuum-dried and silylated with 120 μL N-methyl-N-(trimethylsilyl) trifluoroacetamide (MSTFA) at 27°C for 120 min to separate for GC-MS analysis.

#### 2.3.2 GC-MS Spectral Acquisition

GC-MS analysis was performed using GC/MS-QP2010 Ultra (Shimadzu Inc., Kyoto, Japan) equipped with an electron impact source operating in positive mode with the energy of 70 eV. Separation was achieved on a fused silica capillary column (Rtx-5MS; 30.0 m× 0.25 mm, 0.25 µm, Restek, USA) with a programmed temperature vaporization. The initial oven temperature was held at 70°C for 3 min, ramped to 320°C at a rate of 10°C/min, and finally held at 320°C for 2 min. The injection was performed in split mode (1: 50). Helium (>99.999%) was used as the carrier gas at a constant flow rate of 1.0 mL/min. For mass detection, full scan with a mass range of *m/z* 45-600 was adopted, and the ion source temperature was set at 200°C.

### 2.4 Quality Assurance Procedure

To assure the robustness of analytical system and an acceptable level of data quality for non-targeted metabolomics, pooled QC samples, prepared by mixing equal volumes of each analyzed sample (23), were injected at the beginning of the batch to condition the analytical platform and then almost every six samples to monitor the system. The metabolic features that are detected in < 80% of QC samples (80% rule) and those with a relative standard deviation (RSD), as calculated for the QC samples, of > 30% (RSD 30% rule) were removed (20). The quality assurance procedure was performed to remove metabolic features with poor repeatability.

### 2.5 Statistical Analysis and Pathway Enrichment

The obtained LC-MS and GC-MS raw data files were processed using Profiling Solution version 1.1 (Shimadzu, Japan) for peak detection, matching, and alignment. After filtered by “80% rule” and “RSD 30% rule”, missing values replacement and total ion intensity normalization, the resulting data was imported to SIMCA software 13.0 package (version 13.0; Umetrics, Umeå, Sweden) for multivariate statistical analysis including principal component analysis (PCA) and orthogonal partial least squares discriminant analysis (OPLS-DA). Ions with variable importance in the projection (VIP) exceeding 1.0 in the OPLS-DA model and P-value adjusted by Benjamini-Hochberg method (pFDR) below 0.05 (24) were retained for further identification. Spearman correlation analysis was then applied to explore the correlations between differential metabolites and clinical indices of recipient.

The differential ions generated from LC-MS were tentatively identified based on the public online databases, such as the Human Metabolome Database (http://www.hmdb.ca) and the Metlin database (http://metlin.scripps.edu) and confidently annotated by matching retention time and mass characteristics with those of in-house standards (25); meanwhile, those from GC-MS were characterized by comparing the standard mass fragments in National Institute of Standards and Technology Research Library based on >70% similarity index (26) and confirmed with the characteristics of the authentic standards available in our lab.

To visualize and interpret the metabolic pathways related to PTDM, the differential metabolites were imported into MetaboAnalyst 5.0, which is a free web-based tool that uses the high-quality KEGG metabolic pathway database as the backend knowledge-base. Meanwhile, Cytoscape (http://www.cytoscape.org), a highly popular Java-based open source software tool, was adopted to visualize and analyze metabolite, gene and protein interaction networks. The list of differential metabolites (compound names or KEGG IDs) were first loaded in the Metscape, a plugin for Cytoscape, to construct the compound-reaction-enzyme-gene network. Then, the network centrality parameters, such as degree, betweenness, and centroid value, were computed by CentiScaPe, another plugin for Cytoscape, to extract the core subnetwork.

## 3 Results

### 3.1 Patient Characteristics

A total of 68 recipients, including 32 PTDM subjects and 36 non-PTDM subjects were recruited. The baseline demographic characteristics and clinical data of the two groups were presented in **Table 1**. PTDM group and non-PTDM group were well matched with no significant difference in gender, age, BMI, family history of diabetes, alcohol drinking history, ICU length of stay and hepatitis B infection.

**Table 1:** Clinical characteristics of the recruited PTDM and non-PTDM recipients.

Parameters	Non-PTDM	PTDM	P-value
Total N	36	32	
Sex (male/female)	28/8	22/10	0.290
Age (years)	49.92 ± 10.61	49.06 ± 9.48	0.806
BMI (kg/m^2^)	22.83 ± 3.91	23.53 ± 3.31	0.323
family history of diabetes, n (%)	3	2	0.738
alcohol drinking history, n (%)	4	3	0.809
ICU length of stay (day)	3.19± 3.09	3.13 ± 1.98	0.366
hepatitis B infection, n (%)	25	27	0.826

### 3.2 Metabolomic Analysis

**Figure 1:**
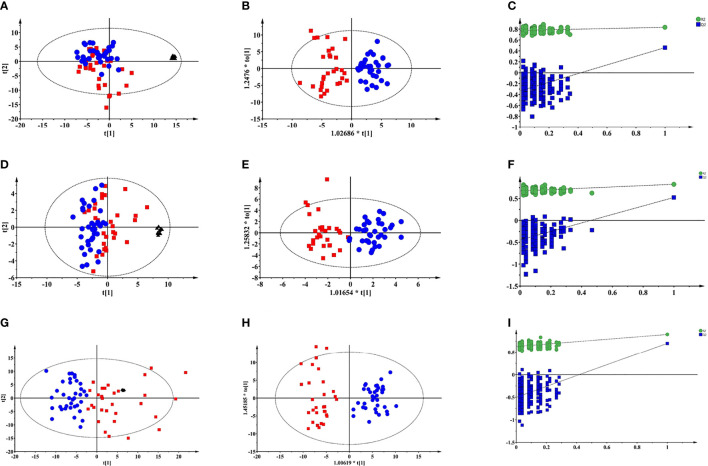
Multivariate modelling of LC-MS and GC-MS data after log transformation and pareto scaling. **(A)** PCA score plot of LC-MS (+) data: R^2^X=0.527, Q^2^ = 0.322; **(B)** OPLS-DA score plot of LC-MS (+) data: R^2^X=0.131, R^2^Y=0.782, Q^2^ = 0.502, CV-ANOVA p value = 1.0e^-10^; **(C)** the 200-permutation test of LC-MS (+) data; **(D)** PCA score plot of LC-MS (-) data: R^2^X=0.613, Q^2^ = 0.255; **(E)** OPLS-DA score plot of LC-MS (-) data: R^2^X=0.165, R^2^Y=0.816, Q^2^ = 0.535, CV-ANOVA p value =2.4 e^-9^; **(F)** the 200-permutation test of LC-MS (-) data; **(G)** PCA score plot of GC-MS data: R^2^X=0.681, Q^2^ = 0.458; **(H)** OPLS-DA score plot of GC-MS data: R^2^X=0.301, R^2^Y=0.899, Q^2^ = 0.701, CV-ANOVA p value =8.0e^-14^; **(I)** the 200-permutation test of LC-MS (+) data:. Blue circles: non-PTDM; red squares: PTDM; black triangles: QC.

Typical total ion chromatograms (TICs) of PTDM and non-PTDM recipients were presented in **Supplementary Figure 1**. However, there was no visual difference in metabolic profiles between PTDM group and non-PTDM group. Therefore, PCA, an unsupervised method of multivariate analysis, was first performed to get an overview of the difference on the metabolic profiles. Outliers were checked using the Hotelling T2 range, adopting 95% and 99% confidence limits for suspect and strong outliers, respectively. Two patients from the PTDM group appeared out of Hotelling’s ellipse at the 99% confidence. These two outliers shared the common feature that their liver function parameters, i.e. aspartate aminotransferase and alanine aminotransferase, were abnormal, and one of them died at the third year post-operation. Since then, the two outliers were removed, multivariate analysis was re-performed. As shown in **Supplementary Figure 2**, except two samples which lay between 95% and 99% Hotelling T2 ellipse, all of the remaining samples lay inside the 95% Hotelling T2 ellipse. Tight clustering of QC samples was observed in PCA score plots (**Figures 1A, D, G**), giving some confidence that the analytical process was running robustly providing reproducible metabolic profiles.

As depicted in PCA score plots (**Figures 1A, D, G**), there was a visual separation between PTDM and non-PTDM groups, indicating metabolic disorders in PTDM. Furthermore, supervised OPLS-DA was introduced to maximize the separation and dig out differential metabolites. All the three models produced a goodness of prediction with Q2 > 0.5 and the differences between R2Y and Q2 <0.3 (27) (**Figures 1B, E, H**). Furthermore, permutation test (200 times) and CV-ANOVA were performed to validate the generated models (28). For the permuted R2 and Q2, all the values were lower than their corresponding original ones, the intercepted value of Q2 in the vertical axis was below 0 and p-values of CV-ANOVA for all models were below 0.05 (**Figures 1C, F, I**), demonstrating high goodness of fit for the generated OPLS-DA models.

**Figure 2:**
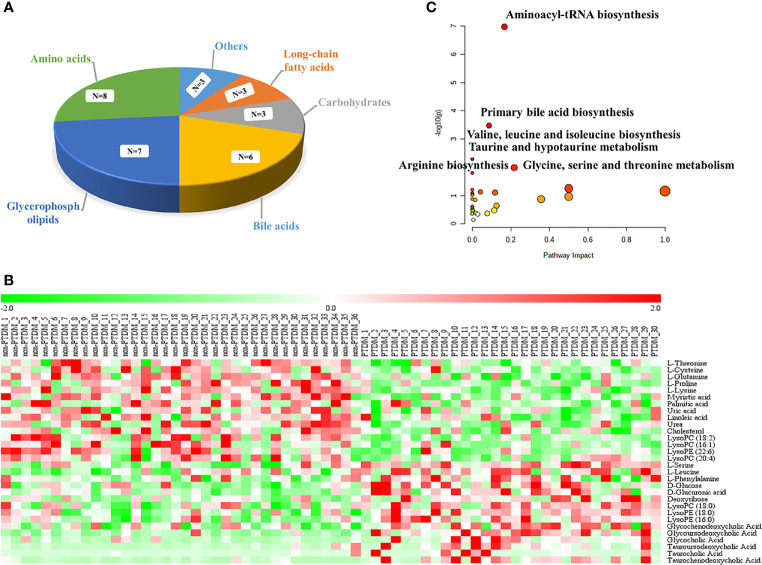
Analysis of the differentially expressed metabolites. **(A)** classification of the 30 identified differentially expressed metabolites; **(B)** Heat map of the differentially expressed metabolites in each group. Rows, samples; columns, metabolites. The red band indicates an increased level of metabolites, while the green band indicates a decreased level of metabolites in PTDM group compared with non-PTDM group; **(C)** Summary of the altered metabolism pathways determined with MetaboAnalyst v. 5.0.

Moreover, the combination of VIP>1 and pFDR<0.05 was applied to screen out the differential metabolites between PTDM and non-PTDM. As a result, a total of 37 differential metabolites (21 from LC-MS, 16 from GC-MS) were identified. Furthermore, Spearman correlation analysis was adopted to explore the correlations between these differential metabolites and fasting plasma glucose. Based on the correlation coefficients, L-valine, LysoPE (20:4), LysoPE (18:2), LysoPC (20:2), LysoPC (18:1), LysoPC (16:0) and LysoPC (14:0) were removed because of the weak correlation (-0.3 < Spearman correlation coefficients < 0.3) (**Supplementary Figure 3**). Finally, a total of 30 differential metabolites (15 from LC-MS, 15 from GC-MS) were retained for further analysis. The detailed information including compound name, molecular formula, retention time and fold change value were shown in **Table 2** (GC-MS data) and **Table 3** (LC-MS data). These 30 differential metabolites annotated six main classes, including eight amino acids, seven glycerophospholipids, six bile acids (BAs), three carbohydrates, three long-chain fatty acids and others (**Figure 2A**). Furthermore, the contents variations of differential metabolites were depicted as a heatmap (rows correspond to metabolites, columns to samples, red and green denote increased and decreased signals in PTDM group compared with non-PTDM group) in **Figure 2B**.

**Table 2:** Differential metabolites identified by GC-MS.

Compound	Datebase ID	Formula	VIP	*p*FDR	Ion RT	Similarity	Fold change
Urea	HMDB00294	CH4N2O	1.34	<0.001	9.29	93	0.89
L-Leucine*	HMDB00294	CH4N2O	1.14	0.001	9.73	74	1.15
L-Serine*	HMDB00187	C3H7NO3	1.51	<0.001	11.03	91	1.20
L-Threonine	HMDB00167	C4H9NO3	1.44	<0.001	11.39	76	0.62
L-Proline*	HMDB00162	C5H9NO2	1.33	<0.001	13.28	93	0.84
L-Cysteine*	HMDB00574	C3H7NO2S	1.15	<0.001	13.71	74	0.69
L-Lysine	HMDB00182	C6H14N2O2	1.35	<0.001	15.59	91	0.86
L-Glutamine	HMDB00641	C5H10N2O3	1.55	<0.001	16.35	84	0.76
Deoxyribose	HMDB03224	C5H10O4	1.16	0.002	17.18	81	1.58
D-Glucose	HMDB00122	C6H12O6	1.21	0.001	17.92	90	1.16
D-Glucuronic acid	HMDB00127	C6H10O7	1.44	<0.001	18.97	83	1.33
Palmitic acid	HMDB00220	C16H32O2	1.17	0.006	19.31	92	0.87
Uric acid	HMDB00289	C5H4N4O3	1.05	0.010	19.78	87	0.88
Linoleic acid	HMDB00673	C18H32O2	1.21	0.001	22.36	77	0.86
Cholesterol	HMDB00067	C27H46O	1.35	<0.001	28.64	92	0.89

* Identification was confirmed with authentic standard.

**Table 3:** Differential metabolites identified by LC-MS.

Rt (min)	Molecular Formula	m/z	ion forms	MS/MS fragment	VIP	*p*FDR	Fold change	Identification
1.17	C9H11NO2	166.0877	[M+H]^+^	120.0864	1.21	0.031	1.19	L-Phenylalanine
15.12	C14H28O2	227.2014	[M-H]^-^	109.1859, 145.8610	1.45	<0.001	0.59	Myristic acid
13.40	C21H44NO7P	454.2942	[M+H]^+^	313.2706, 436.2881	2.12	<0.001	1.44	LysoPE (16:0)
			[M-H]^-^	196.0368, 255.2354				
14.94	C23H48NO7P	482.3258	[M+H]^+^	341.3087, 421.2729, 464.3151	1.86	<0.001	1.26	LysoPE (18:0)
		480.3075	[M-H]^-^	283.2683				
12.58	C24H48NO7P	494.3252	[M+H]^+^	184.0746, 476.3165	1.94	<0.001	0.67	LysoPC (16:1)
		538.3254	[M+HCOO]^-^	253.2237, 478.2988				
13.11	C26H50NO7P	520.3414	[M+H]^+^	184.0755, 443.2782, 502.3330	2.23	<0.001	0.62	LysoPC (18:2)
		564.3337	[M+HCOO]^-^	279.2348, 504.3167				
15.35	C26H5N4O7P	524.3722	[M+H]^+^	184.0750, 311.2981, 447.2860, 506.3643	1.86	0.001	1.22	LysoPC (18:0)
		568.3622	[M+HCOO]^-^	100.5837, 283.2678, 508.3450				
12.95	C27H44NO7P	526.2948	[M+H]^+^	385.2803, 508.2844	1.89	<0.001	0.74	LysoPE (22:6)
		524.2744	[M-H]^-^	196.0423, 283.2474, 327.2220				
13.13	C28H50NO7P	544.3401	[M+H]^+^	184.0779, 485.2655, 526.3357	2.55	<0.001	0.81	LysoPC (20:4)
		588.3329	[M+HCOO]^-^	126.9552, 303.2333, 528.3122				
10.54	C26H43NO5	448.3051	[M+H]^+^	414.3102, 432.3129	1.89	0.001	1.54	Glycochenodeoxycholic Acid
		450.3414	[M-H]^-^	386.3051				
9.10	C26H43NO6	464.2998	[M-H]^-^	295.2011, 364.2687, 402.3076, 446.2918	1.56	0.001	3.67	Glycocholic Acid*
9.28	C26H45NO6S	498.2868	[M-H]^-^	355.2611, 480.2768	1.82	0.003	5.63	Taurochenodeoxycholic Acid*
8.14	C26H45NO6S	498.2869	[M-H]^-^	290.2154, 355.2671, 384.3029, 480.2768	2.12	<0.001	4.30	Tauroursodeoxycholic Acid
8.19	C26H45NO7S	514.2822	[M-H]^-^	515.2866	1.12	0.028	5.14	Taurocholic Acid*
9.24	C26H43NO5	448.3051	[M-H]^-^	386.3108, 449.3126	2.54	<0.001	2.72	Glycoursodeoxycholic Acid

* Identification was confirmed with authentic standard.

### 3.3 Altered Pathways Related to PTDM

**Figure 3:**
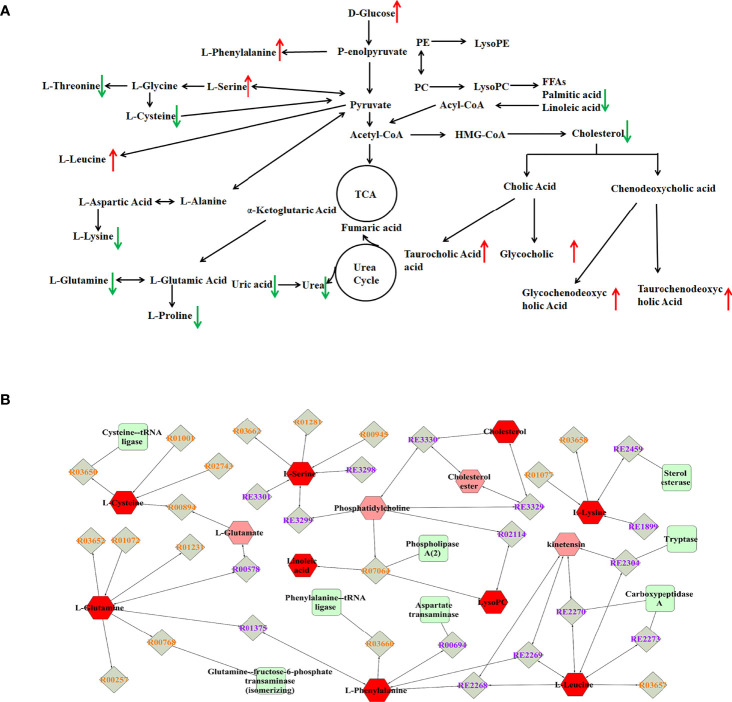
Analysis of potential biomarkers and related pathways. **(A)** Schematic overview of metabolic pathways based on the differentially expressed metabolites. The metabolites indicated with red or green arrows represent increased or decreased levels, respectively, in PTDM group. **(B)** The compound-reaction-enzyme-gene subnetwork. Red hexagons represent the identified differentially expressed metabolites. Green squares represent enzymes which might regulate the identified metabolites. Grey diamonds represent reactions catalyzed by those enzymes.

To explore potential metabolic pathways involved in PTDM, the differential metabolites were imported into MetaboAnalyst for functional enrichment analysis and network topology analysis. Results (**Figure 2C**) indicated that aminoacyl-tRNA biosynthesis, valine, leucine and isoleucine biosynthesis, primary bile acid biosynthesis, taurine and hypotaurine metabolism, glycine, serine and threonine metabolism, arginine biosynthesis with p-value less than 0.05 were the critical disturbed pathways involved in progression of PTDM (29, 30). To clearly elucidate the possible underlying mechanism of PTDM, a hypothetical metabolic network was reconstructed by using these differential metabolites, with the direction of the content change labeled (**Figure 3A**).

Moreover, the MetScape plugin for Cytoscape was used to construct the compound-reaction-enzyme-gene network based on the 30 differential metabolites (31). Among them, LysoPCs, and LysoPEs were regarded as category IDs. Additionally, for some metabolites, such as tauroursodeoxycholic acid (TUDCA), glycoursodeoxycholic acid (GUDCA) and glycochenodeoxycholic acid (GCDCA) were not retrieved in the MetScape plugin or KEGG database. Hence, there were 542 nodes containing 154 compounds, 112 reactions, 87 enzymes and 189 genes in the network (**Supplementary Figure 4**). Then, the node centrality indexes, namely degree, betweenness, and centroid value, were calculated to extract the core subnetwork displaying a critical role in the process of PTDM (32). As shown in **Figure 3B**, nine metabolites, namely linoleic acid, L-leucine, L-glutamine, L-phenylalanine, L-cysteine, cholesterol, L-serine, L-lysine, and LysoPC, were selected as hub metabolites. Meanwhile, eight enzymes, such as aspartate transaminase, phospholipase A2 and tryptase, were involved in this subnetwork.

## 4 Discussion

As a frequent metabolic complication, PTDM seriously affects the life quality and long-term survival of recipients. However, data on the mechanism of PTDM are scarce. Beyond this, the treatment is based on expert experience rather than research-based evidence at the current stage. Metabolomics, encompassing the comprehensive and systematic profiling of multiple metabolites, is a promising approach to provide an understanding of physiological and pathological status of the living organism. Nevertheless, reports on the adoption of metabolomics to describe the metabolic profiles and pathways involved in PTDM have not been published. In this study, for the first time to our knowledge, the metabolic profiles and pathways involved in PTDM were explored by the integrated LC-MS and GC-MS based-metabolomics, which aimed to provide novel insights into the underlying pathophysiological mechanisms of PTDM. All individuals enrolled in our study were free of diabetes pre-transplantation and matched on gender, age, BMI, family history of diabetes, alcohol drinking history, ICU stay length and hepatitis to minimize confounding factors.

The integrated untargeted metabolomics revealed that 30 significantly changed metabolites, among which 15 decreased and 15 increased, possibly contributed to the development of PTDM. Based on their chemical structure, these significantly changed metabolites mainly belongs to the classes of amino acids, bile acids, glycerophospholipids and others.

Our findings highlighted several amino acids, particularly the branched-chain amino acids (BCAAs) and aromatic amino acids (AAAs), were noteworthy and might be served as biomarkers of PTDM. BCAAs (leucine, isoleucine and valine) and AAAs (tyrosine, phenylalanine and tryptophan) have been proven to be potential contributors to the development of insulin resistance and diabetes in both humans (33) and rodent models (34). A nested case-control study in the Framingham Offspring Study comprising 2422 normoglycemic individuals followed for 12 years indicated that plasma levels of three BCAAs (isoleucine, leucine, valine) and two AAAs (tyrosine and phenylalanine) exhibited highly significant associations with the future development of T2DM (35). Furthermore, a meta-analysis focusing on dietary BCAAs intake and T2DM showed that oral BCAAs supplementation is positively associated with T2DM risk (36). The same result was found in double AAAs intake to mice (37). Our results also suggested L-phenylalanine and L-leucine increased in participants with PTDM. Since BCAAs and AAAs (expect tyrosine) are essential amino acid which must be obtained from the diet, their elevated circulating levels might be the result of excess intake and/or disruption of their catabolic process. However, epidemiological results are controversial, with some indicating that a diet high in BCAAs were positively associated with circulating levels (38), while others not (35). Since then, we inferred that the elevated circulating levels of BCAAs might arise from the hindrances to their downstream catabolism. Unlike most amino acids, whose catabolism take place in the liver, BCAAs are initially catabolized by branched-chain-amino-acid aminotransferase (BCAT) in extrahepatic tissues (such as skeletal muscle) to form branched chain α-keto acids (BCKAs) and then by branched chain α-keto acid dehydrogenase (BCKD), the rate-limiting enzyme in BCAA catabolism. Zhou M et al. (39) revealed that the enhancement of BCKD activity by administration of BT2 to BCKD deficiency obese (ob/ob) mice reduced the abundance of BCAAs and BCKAs, resulting in markedly attenuated insulin resistance. The BCAA catabolism was suggested as a potential therapeutic target for insulin resistance and T2DM. In addition, recent work revealed that elevated circulating BCAAs levels correlated with intestinal microbiota dysbiosis of the host. *Prevotella copri* and *Bacteroides vulgatus* were proven to be the main species associated with the biosynthesis of BCAAs and insulin resistance (40). Gavage with *Prevotella copri* would induce insulin resistance, aggravate glucose intolerance and augment circulating levels of BCAAs (38), while Gavage with *Bacteroides vulgatus* exerted the opposite effect (41). Similar to previous studies, the levels of L-leucine and L-phenylalanine were significantly higher in PTDM recipients than in non-PTDM ones, which might be due to BCAAs catabolism hindrance and/or host intestinal microbiota dysbiosis.

Significant variations in specific BAs species were found in our study. Simply put, the levels of taurocholic acid (TCA), TUDCA, taurochenodeoxycholic acid (TCDCA), glycocholic acid (GCA), GUDCA and GCDCA were significantly higher in PTDM receipts than in non-PTDM ones. As the most frequent etiology of liver transplantation (76.5%) in our study, hepatitis B virus has been proven to alter the expression of CYP7A1, a key enzyme involved in bile acid synthesis (42). Thus, the disturbance in BA profiles has been repeated observed in hepatitis B virus-infected patients for decades (43, 44). To minimize the influence from this confounding factor, case and control subjects were well-matched in hepatitis B virus infection. What’s more, in terms of aspartate aminotransferase, alanine aminotransferase, hepatic function of liver transplant recipients normally recovered within a few days, which was consistent with previous research (45). Two participants with hepatic disfunction were excluded from data analysis. Over the last few decades, BAs have attracted considerable attention in the field of diabetes, obesity, nonalcoholic fatty liver disease and so on. BAs are synthesized in hepatocytes and then undergo enterohepatic circulation with six to eight times per day in humans. Thus, BAs are detected at relatively lower levels in plasma compared with them in the liver, bile and intestine. In human, most bile acids are conjugated to glycine (G) and taurine (T) at a ratio of about 3:1. To date, it is still uncertain whether and what circulating BAs alter in patients with T2DM. For instance, a cross-sectional study including 224 T2DM patients and 102 nondiabetic individuals indicated that patients with T2DM possessed increased plasma levels of TCDCA, GCDCA, deoxycholic acid (DCA), taurodeoxycholic acid (TDCA) and glycodeoxycholic acid (GDCA), and decreased levels of CA and TCA (46). Another case-control study of age- and gender-matched T2DM versus control demonstrated elevated levels of TCA, TDCA, GDCA and DCA in T2DM subjects (47). Furthermore, a nested case-control study of 1,707 matched T2DM-control subject pairs within the China Cardiometabolic Disease and Cancer Cohort Study showed that conjugated primary BAs (GCA, TCA, GCDCA and TCDCA) and secondary BA (TUDCA) were positively related with T2DM risk, while unconjugated BAs (CA, CDCA and DCA) were inversely associated with T2DM risk (48). Accordingly, the currently human studies provided conflicting results, with some reporting certain BAs species increased in T2DM and others reporting those decreased in T2DM. Beyond this problem, considering the relatively small number of participants, the variation of BAs in PTDM recipients need to be verified in a large cohort.

What’s more, we found a series of LysoPCs and LysoPEs expressed differentially in PTDM recipients. The concentration of lysoPCs in plasma, up to 100μM in healthy subjects (49), is much higher than that of lysoPEs. In plasma, LysoPCs, representing 5%-20% of total phospholipids, are mainly formed by lecithin-cholesterol acyltransferase (LCAT) in the process of transferring fatty acyl residues in sn-2 position of phosphatidylcholine to free cholesterol for the formation of cholesteryl esters, or by endothelial lipase, or by direct hepatic secretion (50). The alterations of LysoPCs species linked to T2DM have been widely studied. Significant lower levels of LysoPC (18:2), LysoPC (18:1), LysoPC (18:0), and LysoPC (17:0) were found in T2DM and impaired glucose tolerance (IGT) cohort in the population-based Cooperative Health Research in the Region of Augsburg (KORA) study. Among them, LysoPC (18:2) served as a predictor for T2DM, which was independently confirmed in the European Prospective Investigation into Cancer and Nutrition (EPIC)-Potsdam study (51). A global lipidomics analysis of 293 Chinese individuals has also shown that LysoPC (18:0), LysoPC (18:1), and LysoPC (18:2) were all negatively correlated with HOMA-IR (52). Our finding was in agreement with above findings that LysoPC (18:2) exhibited significantly lower level in PTDM than non-PTDM recipients. Several other studies have reported inconsistent findings, i.e. diabetic men exhibited higher levels of centain LysoPCs, including LysoPC (14:0), LysoPC (16:1), LysoPC (18:1), LysoPC (22:6), LysoPC (20:5) and LysoPC (18:3), but not including LysoPC (18:2), LysoPC (16:0) and LysoPC (18:0) (53). Our study exhibited inverse change trends of different LysoPC species with increased expression in LysoPC (18:0) and decreased expression in LysoPC (18:2), LysoPC (16:1) and LysoPC (20:4), which might be due to the opposite effects of saturated and unsaturated acyl LysoPC. Park JY et al. (54) reported that lysoPC and lysoPE species containing unsaturated fatty acids were associated with an increased risk of coronary artery disease, whereas those containing saturated fatty acids were associated with a decreased risk. Saturated LysoPCs, such as LysoPC (16:0), are a potent inflammatory mediator, while polyunsaturated acyl LysoPCs, including LysoPC (20:4) and LysoPC (22:6), can serve as an anti-inflammatory lipid mediator and inhibit the inflammation induced by saturated LysoPCs (55). In mouse models, Yea K et al. (56) have reported that the blood glucose lowering effect of LysoPCs were found to be sensitive to variations in lysoPC acyl chain length, which may elucidate the divisive findings in our study. Therefore, lysoPCs play a complex role in T2DM, especially special type of T2DM like PTDM, which needs further work to clarify.

Our study recruited liver transplant recipients to address the “real-world” problem in PTDM. The metabolomic results help to give a new sight in the mechanism of PTDM. Since the analyzed sample size was small, we speculate that PTDM might be associated with the perturbation in amino acids, bile acids and glycerophospholipids. This hypothesis provides possible research direction in the field of PTDM. In addition, a major limitation of plasma metabolomics is that all of the differential metabolites are detected in plasma, their actual origins are unclear. Further studies should investigate the highlighted pathways in relevant tissues (such as muscle and liver) and their relations to PTDM for a comprehensive understanding of its underlying mechanism.

## 5 Conclusion

In summary, the integrated LC-MS and GC-MS based-metabolomics was adopted to dig out differentially changed metabolites associated with PTDM. A total of 30 metabolites (15 from LC-MS, 15 from GC-MS) significantly altered in PTDM recipients were identified. Findings indicated that alterations in plasma metabolites, particularly amino acids, BAs and LysoPCs may contribute to the progression of PTDM. Our study offered a novel insight into the pathological mechanism of PTDM. Further studies are needed to verify these findings and to unravel the underlying mechanisms involved in PTDM.

## Data Availability Statement

The original contributions presented in the study are included in the article/**Supplementary Material**. Further inquiries can be directed to the corresponding authors.

## Ethics Statement

The studies involving human participants were reviewed and approved by the Ethics Committee of the Affiliated Drum Tower Hospital of Nanjing University Medical School (No. 2020-053-01). Written informed consent for participation was not required for this study in accordance with the national legislation and the institutional requirements.

## Author Contributions

MW, HZ, and JW designed the study protocol. MW, JX, and NY acquired the data and interpreted the results. JX and TZ wrote the original draft. MW, NY, and TZ recruited subjects. HZ and JW supervised the project and revised the original draft. All authors contributed to the article and approved the submitted version.

## Funding

This study was supported by the Natural Science Foundation of Jiangsu Province (No. BK20180129; BK20190122), Aosaikang Hospital Pharmacy Foundation of JiangSu Pharmaceutical Association (No. A201906) and Nangjing Medical Center for Clinical Pharmacy.

## Conflict of Interest

The authors declare that the research was conducted in the absence of any commercial or financial relationships that could be construed as a potential conflict of interest.

## Publisher’s Note

All claims expressed in this article are solely those of the authors and do not necessarily represent those of their affiliated organizations, or those of the publisher, the editors and the reviewers. Any product that may be evaluated in this article, or claim that may be made by its manufacturer, is not guaranteed or endorsed by the publisher.
