# Supplementary material for: Insight Into the Metabolomic Characteristics of Post-Transplant Diabetes Mellitus by the Integrated LC-MS and GC-MS Approach- Preliminary Study
Source: Front Endocrinol (Lausanne). 2022 Jan 18;12:807318. doi: 10.3389/fendo.2021.807318 (PMC8805207; doi:10.3389/fendo.2021.807318)
Supplement: Supplementary file 1 [file Table_1.docx]

Supplementary Material


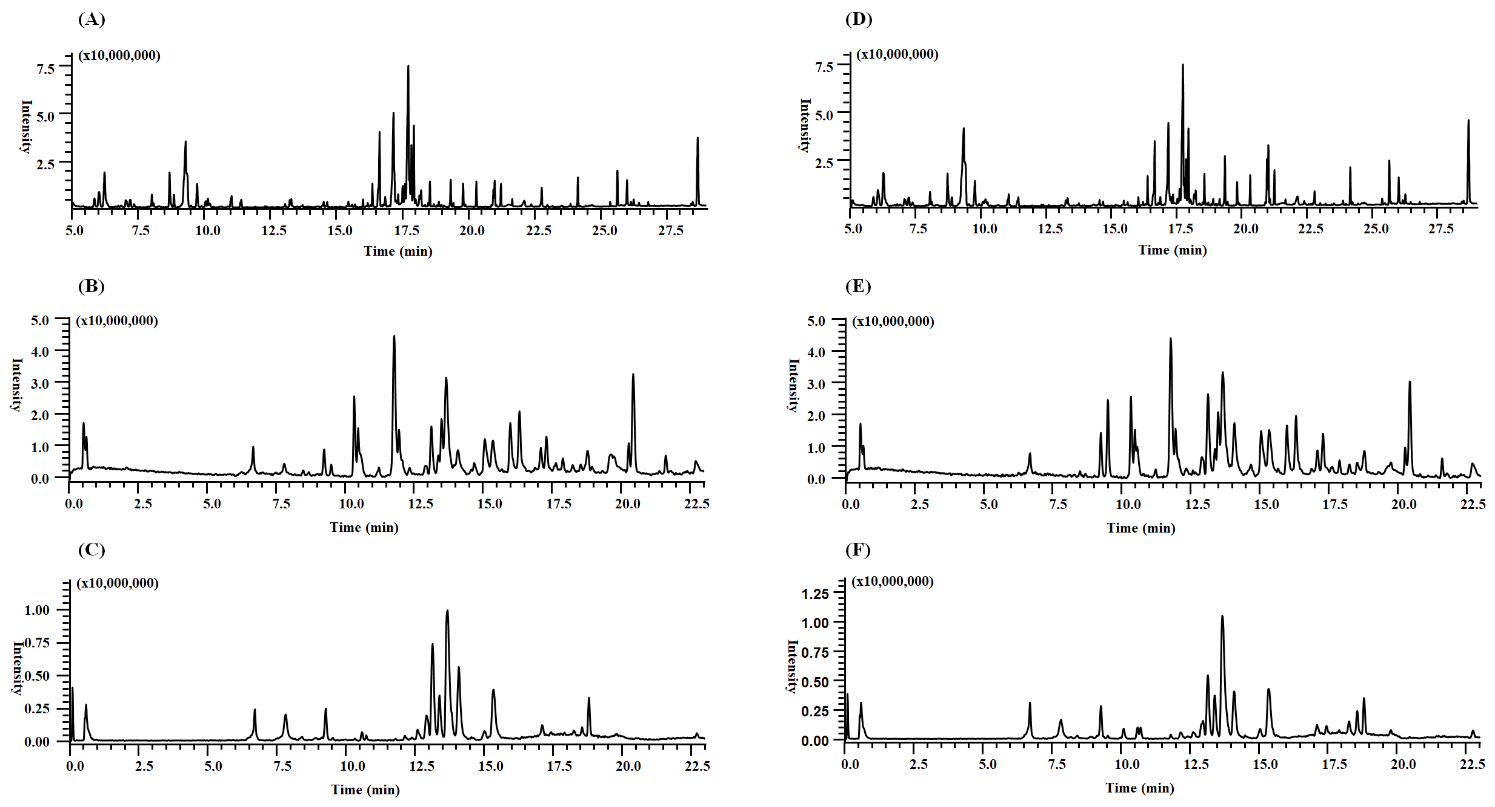


FIGURE S1. Representative TICs of recipient plasma. TICs of non-PTDM (A) and PTDM (B) recipients derived from GC-MS; TICs of non-PTDM (C) and PTDM (D) recipients derived from LC-MS positive mode; TICs of non-PTDM (E) and PTDM (F) recipients derived from LC-MS negative mode.


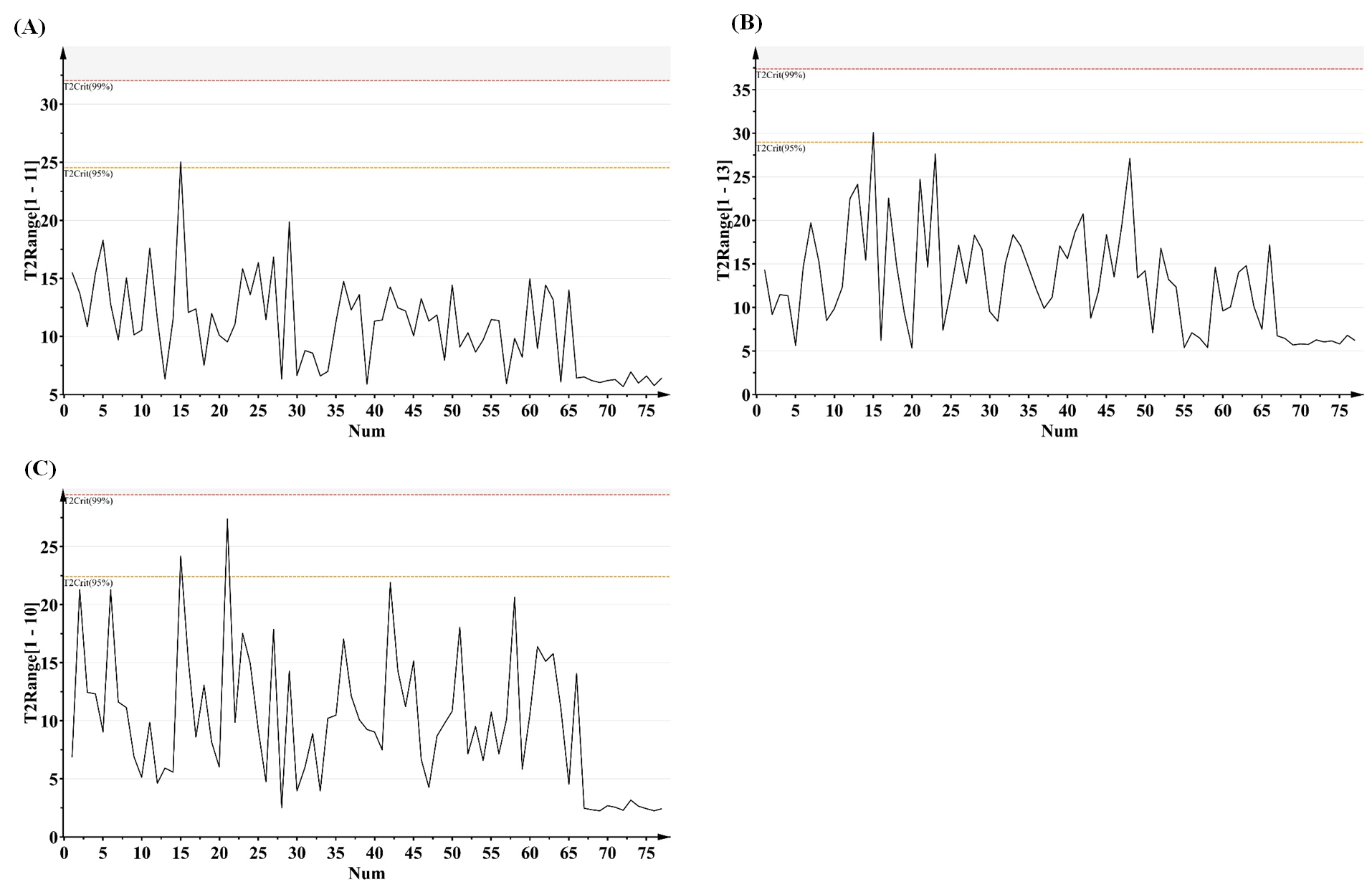


FIGURE S2. Hotelling T2 range plot. (A) LC-MS (+); (B) LC-MS (-); (C) GC-MS.


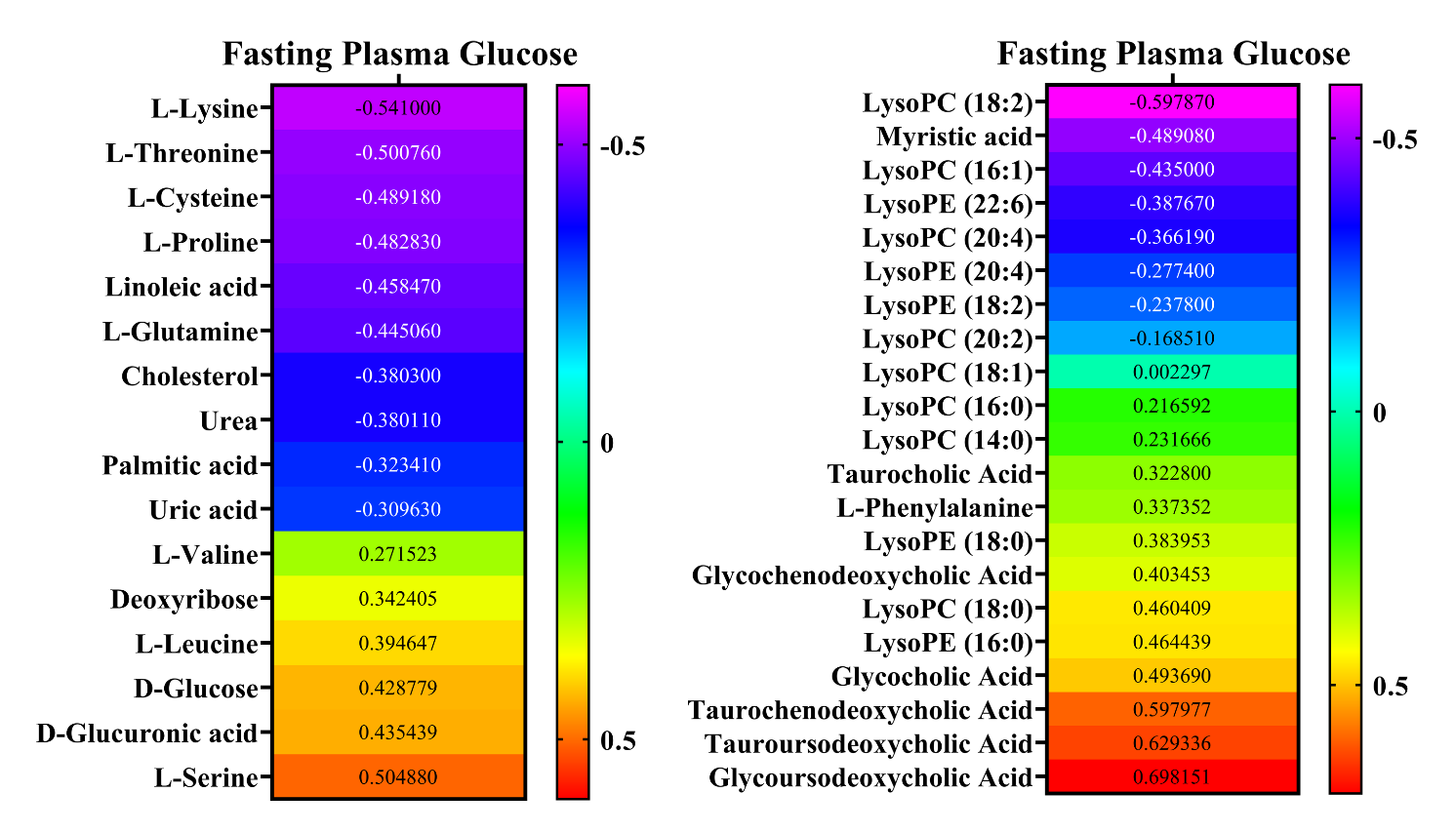


FIGURE S3. Spearman correlation analysis of differential metabolites and fasting plasma glucose.


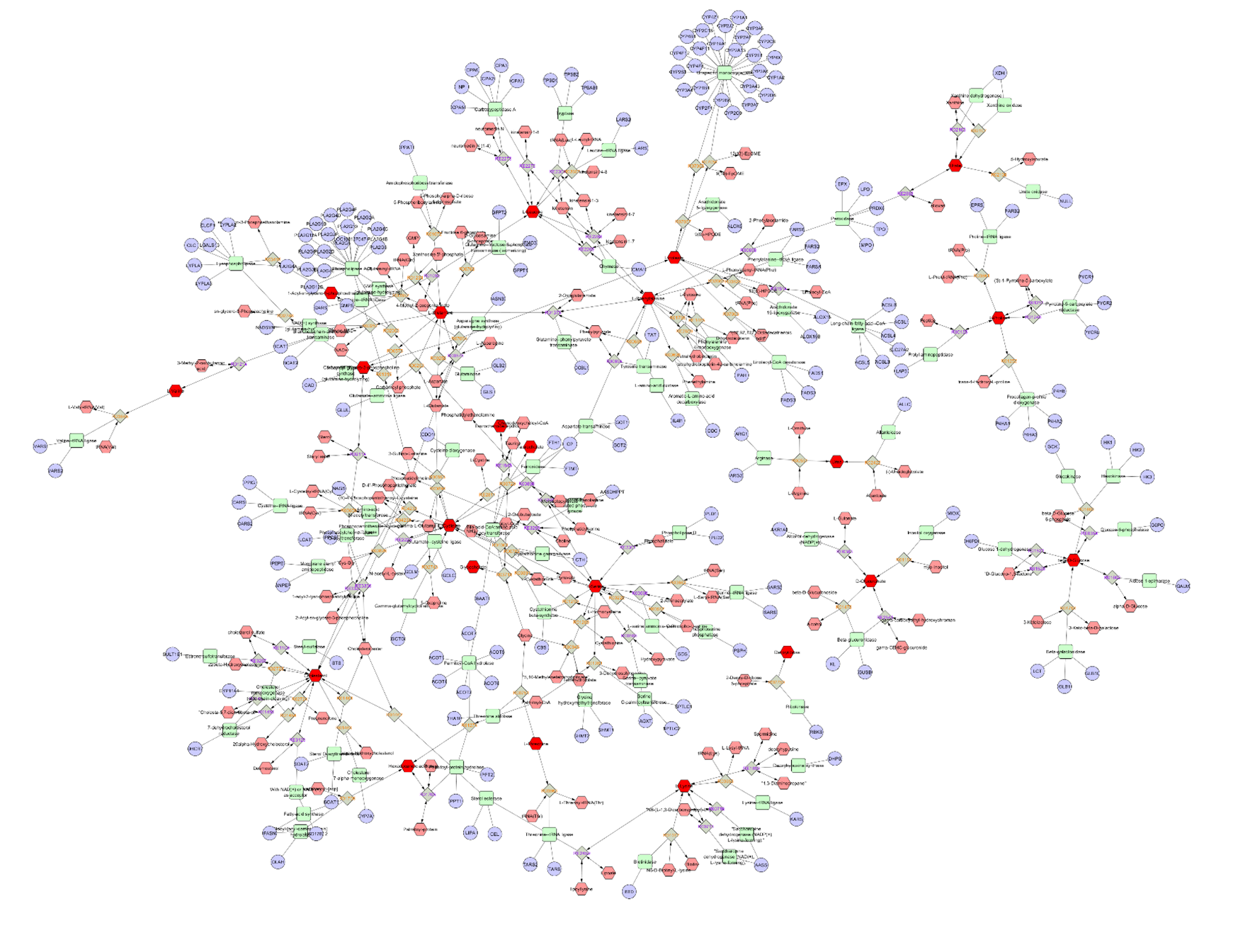


FIGURE S4. The compound-reaction-enzyme-gene network of differential metabolites associated with the development of PTDM.
